# Enhancing psychiatry education: effectiveness of a psychodynamic psychotherapy module for borderline personality disorder for psychiatry residents

**DOI:** 10.3389/fpsyt.2026.1712435

**Published:** 2026-07-02

**Authors:** Petrin Redayani Lukman, Tjhin Wiguna, Diantha Soemantri, Sri Linuwih Menaldi, Sylvia Detri Elvira, Limas Sutanto, Tuti Wahmurti A. Sapiie, Aria Kekalih, Reina Rahma Noviasari, Hukma Shabiyya Rizki, Kharisma Zatalini Giyani, Nik Ruzyanei Nik Jaafar

**Affiliations:** 1 Doctoral Program in Medical Sciences, Faculty of Medicine, Universitas Indonesia, Jakarta, Indonesia; 2 Department of Psychiatry, Faculty of Medicine, Universitas Indonesia, Dr. Cipto Mangunkusumo General Hospital, Jakarta, Indonesia; 3 Department of Medical Education, Faculty of Medicine, Universitas Indonesia, Jakarta, Indonesia; 4 Department of Dermatology and Venereology, Faculty of Medicine, Universitas Indonesia, Dr. Cipto Mangunkusumo General Hospital, Jakarta, Indonesia; 5 Division of Psychoanalysis in Psychiatry, Psychotherapy Section, Indonesian Psychiatric Association, Jakarta, Indonesia; 6 Department of Psychiatry, Faculty of Medicine, University of Padjadjaran, Bandung, Indonesia; 7 Department of Community Medicine, Faculty of Medicine, Universitas Indonesia, Jakarta, Indonesia; 8 Department of Psychiatry, Universiti Kebangsaan Malaysia, Kuala Lumpur, Malaysia

**Keywords:** borderline personality disorder, effectivity, Indonesian psychiatry residents, learning module, learning outcome, online learning, psychodynamic psychotherapy

## Abstract

**Background:**

Psychodynamic psychotherapy is the treatment of choice for borderline personality disorder (BPD); however, psychiatric residents frequently report difficulty in applying it, partly due to the lack of structured training models. This study developed and evaluated the effectiveness of psychodynamic psychotherapy learning modules for BPD among Indonesian psychiatry residents.

**Methods:**

A quasi-experimental pre-/post-test control group study using mixed methods was conducted across nine psychiatric residency programs in Indonesia. Thirty-four residents were recruited, of whom 33 completed the study. Learning outcomes were assessed using multiple-choice questions and the Psychodynamic Formulation Competency Assessment Scale (PF-CAS) and Practical Competency Assessment Scale (PC-CAS). The module program was evaluated by the participants using the Indonesian version of the Kirkpatrick Level 1 questionnaire.

**Results:**

The intervention group showed significantly greater improvement in psychodynamic formulation skills (PF-CAS) than the control group (p < 0.001). The multiple-choice scores improved in both groups, with no significant between-group differences. The intervention group showed a numerically greater improvement in Practical Skills (PC-CAS) than the control group, although the difference was not statistically significant. Participants’ feedback was highly positive, emphasizing the usefulness of psychodynamic formulation training, psychotherapy protocols, and supervision.

**Conclusion:**

Implementation of the psychodynamic psychotherapy for BPD Learning Module enhanced competencies in the cognitive and affective domains and showed promising trends in practical skills. This positive reception highlights its feasibility and potential benefits as part of the residency training curriculum.

## 1 Introduction

Borderline personality disorder (BPD) is one of the most disabling mental disorders worldwide. With interpersonal dysfunction proposed as the central pathology, individuals with BPD constantly alternate between seeking connections with others and mistrusting them because of an extreme fear of rejection and abandonment. Thus, they frequently experience intense and stormy inter-personal relationships (1, 2). The chronic nature of the disease is accompanied by perpetual feelings of emptiness, lack of direction in one’s life, and a markedly higher rate of suicide compared to other personality disorders (3–5). Suicidal behavior is observed in 75% of individuals with BPD and approximately 10% commits suicide (4). Although BPD has a fairly low prevalence in the general adult population, it constitutes a significant proportion of both psychiatric outpatient and inpatient diagnoses, with prevalence rates of 11-12% and 22%, respectively (6). Moreover, individuals with BPD are predominantly present in emergency settings during a state of crisis, reflecting the severity of the pathology (7).

Psychotherapy is the preferred treatment for patients with BPD. Psychoanalytically oriented psychodynamic psychotherapy focuses on improving an individual’s understanding of unconscious conflicts that impact their interpersonal and affective difficulties. Although it is effective in improving symptoms and interpersonal functioning, performing psychodynamic psychotherapy for individuals with BPD is often difficult (6). Individuals with BPD may express intense emotional states toward the therapy process or the therapists themselves, which then induce negative countertransference reactions, such as anger or feelings of incompetence in the therapist (8, 9). Therapists often feel undertrained and inadequately equipped to treat individuals with BPD (10).

Several studies have highlighted the importance of a specialized training model for psychodynamic psychotherapy in BPD (11, 12). Such models might be especially beneficial for psychiatry residents who typically receive only general psychotherapy training, which might not adequately supply them with specific knowledge of the pathology and treatment of BPD (11). Although learning models for the general principles of BPD treatment currently exist, there have not been specific training models that focused on psychodynamic psychotherapy practice that is deemed applicable for residents (11, 12). The Good Psychiatric Management (GPM) developed by Unruh and Gunderson only focused on the general principles of managing the case of BPD and did not focus on psychodynamic psychotherapy (11). While a learning model for Dialectical Behavioral Therapy (DBT) exists, DBT could be difficult to implement in non-specialized settings and needed a more comprehensive approach. Meanwhile, psychodynamic psychotherapy is a core competency that is required to be achieved by psychiatry residents in Indonesia (13). In 2020, a survey was conducted by the Psychotherapy Section of the Indonesian Psychiatric Association aimed at 22 teaching staff psychiatrists across Indonesia revealed that several residents reported difficulty in performing psychodynamic psychotherapy with individuals with BPD, and the lack of a structured curriculum for psychodynamic psychotherapy for BPD might contribute to this issue (14). Considering this, developing a specific training model on psychodynamic psychotherapy for BPD for psychiatry residents might bring significant benefits for resident education with the end/ultimate/higher goal of improving patient care for individuals with BPD.

Our team developed a specific learning module for psychodynamic psychotherapy for BPD among psychiatry residents and examined its effectiveness by implementing it in a group of psychiatry residents throughout Indonesia. The module was evaluated using the Kirkpatrick model of training program evaluation, namely Levels 1 and 2, which encompass the reaction to the module and the learning outcome of the module, respectively. Residents’ reactions to the module were evaluated using the adapted Indonesian version of the Kirkpatrick Level 1 questionnaire, while the learning outcome of the improvement of competency in performing psychodynamic psychotherapy for BPD was evaluated on the cognitive, affective, and psychomotor domains. Competency was measured using the psychodynamic formulation competency assessment scale (PF-CAS) to assess the skill of creating a psychodynamic formulation by evaluating the therapists’ written psychodynamic formulation and the practical competency assessment scale (PC-CAS) in psychodynamic psychotherapy for BPD to assess therapists’ skills in performing psychodynamic psychotherapy for patients with BPD in real-life clinical settings by assessing a video recording of a psychotherapy session or by direct observation of a session. PF-CAS and PC-CAS were original scales that we had previously developed in an attempt to create more structured and objective scoring scales to assess the competency of psychodynamic psychotherapy, since the existing international scales were not made in a scoring rubric format (15).

## 2 Materials and methods

### 2.1 Study design

This mixed qualitative-quantitative study was conducted by the Faculty of Medicine, Universitas Indonesia, from August 2023 to January 2024, with the study population being psychiatry residents at multiple universities across Indonesia. This quantitative study included a quasi-experimental design of a pre-test post-test control group to evaluate learning outcomes. The qualitative study included a descriptive analysis design to obtain the distribution of measurement scores based on the Kirkpatrick Level 1 (reaction) questionnaire from psychiatric resident participants after receiving a newly developed Psychodynamic Psychotherapy for Borderline Personality Disorder (PP-BPD) learning module for Indonesian psychiatric residents.

### 2.2 Ethics statement

This study was approved by the Health Research Ethics Committee of the Faculty of Medicine at Universitas Indonesia (protocol number: 22-10-1201; KET-1074 UN2.F1, ETIK/PPM.00.02, 2022). Written informed consent was obtained from all participants before their inclusion in the study.

### 2.3 Participants

We used a stratified random sampling technique to recruit participants across nine psychiatric residency training institutions in Indonesia: University of Padjadjaran, University of Sebelas Maret, Universitas Indonesia, Universitas Udayana, Universitas Sumatera Utara, Universitas Airlangga, Universitas Diponegoro, and Universitas Hasanuddin. These nine institutions were randomly assigned to two groups, with four institutions allocated to the control group and five to the intervention group. Subsequently, stratified random sampling was performed within each institution to ensure that each group comprised 17 participants. Sample size for each institution was calculated using the proportional allocation formula to ensure that the sample size in each institution was proportional to the number of psychiatry residents in the institution.

Sample size determination was guided by Creswell’s recommendation, which states that a minimum of 15 participants per group is required for experimental research in education (16). To account for potential attrition, a 10% dropout adjustment was applied, resulting in a final target sample size of 17 participants per group (17). Consequently, the total sample size was 34.

The inclusion criteria were psychiatric residents in their 6th or 7th semester of training who had completed the basic psychotherapy module and agreed to participate in the study until its completion. Participants were required to complete all assigned tasks and provide informed consent. Participants were classified as dropouts if they failed to complete the assigned tasks during training and/or had an attendance rate below 80%.

### 2.4 Procedures and analysis

The PP-BPD learning module for Indonesian psychiatric residents was developed previously, along with a suite of validated assessment instruments, including a set of multiple-choice questions, the PF-CAS, and the PC-CAS. This study employed Kirkpatrick’s framework to assess Level 1 (reaction) and Level 2 (learning) outcomes using instruments that had undergone prior validation to comprehensively evaluate the module’s efficacy.

The development and full content of the PP-BPD learning module was described in a prior study (18). The PP-BPD module was an online course encompassing 12 weekly and bi-weekly synchronous sessions which was delivered across three months. The module teaches the specific competency of performing psychodynamic psychotherapy for BPD along with the related theories and principles. It is composed of three sequential submodules: (1) Borderline Personality Disorder, (2) Basic Principles of Psychodynamic Psychotherapy, and (3) The Practice of Psychodynamic Psychotherapy for Patients with Borderline Personality Disorder. Five types of learning materials were developed for the module: (1) three coursebooks for each submodule, (2) four lecture slides, (3) two video recordings of psychodynamic psychotherapy sessions, (4) a document of psychodynamic formulation of a patient with BPD, and (5) a document of process notes of psychodynamic psychotherapy sessions. Synchronous sessions between the tutor and the participants were held using the teleconference software program Zoom Workplace. Learning materials and recording of the sessions were uploaded to an online Learning Management System (LMS) so participants could learn it asynchronously on their own time.

Submodule 1 consisted of 2 sessions of interactive lecture. Submodule 2 consisted of 3 sessions of interactive lecture, case discussion, video discussion, and an assignment of formulating a psychodynamic formulation from a case illustration. Submodule 3 consisted of 7 sessions of interactive lecture, case discussion, video discussion, and three assignments of assessing a video recording of a psychodynamic psychotherapy session, formulating a psychodynamic formulation, and writing process notes of psychodynamic psychotherapy sessions. Discussion and feedback sessions were held for each learning activities.

The PF-CAS and PC-CAS were used to assess the participants’ learning outcome before and after undergoing the module. The development of these scales along was described in a prior study (15). PF-CAS consists of 6 evaluation items while the PC-CAS consists of 12 evaluation items. The scales were designed to be used by an assessor to evaluate another psychiatrist or a psychiatry resident’s performance on creating a written psychodynamic formulation for PF-CAS, and on conducting psychodynamic psychotherapy for BPD patients for PC-CAS. The scales are formed as evaluation rubrics with items quantitatively scored on a scale from 0 to 3. Each score is equipped with a description to assist the assessor in determining the appropriate score for the residents’ performance. The final score of each scale is obtained by totaling all the item scores and converting them into a numeric score ranging from 0 to 100. The scales have been validated and obtained good validity, with scale-level content validity index average (S-CVI/Ave=0.981) for PF-CAS and S-CVI/Ave=1.00 for PC-CAS. The interrater reliability of the scales was assessed using the intraclass correlation coefficient (ICC) with a two-way random-effects single-measures consistency model. Three raters assessed four documents on psychodynamic formulations using the PF-CAS, and a different group of three raters assessed four videos of psychiatric residents performing psychodynamic psychotherapy on patients with BPD using the PC-CAS. All groups of raters received training on how to use the scales. The ICC values for the PF-CAS and PC-CAS in psychodynamic psychotherapy for BPD were 0.879 and 0.727 respectively, indicating good reliability of the scales.

The Kirkpatrick Level 1 (Reaction) questionnaire was adapted into Indonesian language from the Voting Assistance Officers Training Evaluation Form, developed by Research and Development (RAND) National Defense Research Institute (NDRI) (19). The questionnaire consisted of 18 statements to be scored on 5-point Likert scale items from 1 (= strongly disagree) to 5 (= strongly agree) evaluating the participants’ response to the module. Furthermore, it included 3 open-ended questions about the participants’ opinion on the important things they have learned from the module as well as feedback for the module. The questionnaire attained sufficient content validity (S-CVI/Ave=0.989) and reliability (Cronbach’s Alpha = 0.954) values.

The methodological framework for this effectiveness study consisted of several sequential phases: recruitment of study participants; baseline competency assessment via a pre-test; structured implementation of the module tailored to the control and intervention groups; post-intervention evaluation through a post-test; and data analysis. Instructional delivery and data collection were conducted remotely via a Learning Management System (LMS).

### 2.5 Study procedure

#### 2.5.1 Participant recruitment

**Figure 1:**
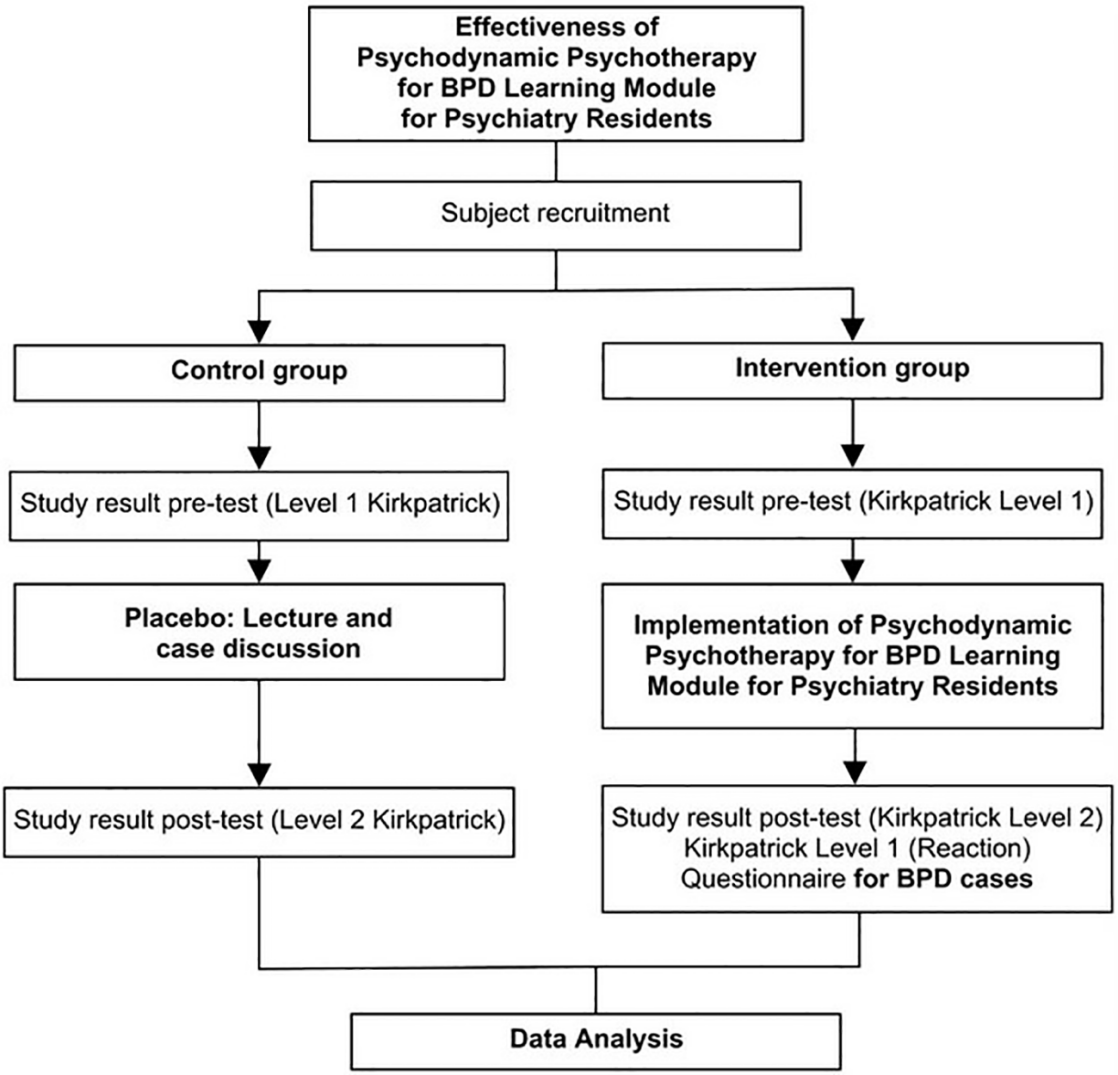
Study procedure.

**Figure 1** shows the flow of the study. A total of 34 psychiatry residents from nine psychiatry residency training institutions in Indonesia were recruited for this study. These institutions were randomly assigned to either the control or intervention group. All participants provided informed consent before participation.

#### 2.5.2 Baseline assessment (pre-test)

Before module implementation, participants in both the control and intervention groups underwent a pretest to assess their baseline competencies across multiple learning domains.

Cognitive Domain Evaluation: Participants completed a set of multiple-choice questions test.

Cognitive and Affective Domain Evaluation: Participants submitted a written psychodynamic formulation report evaluated using the PF-CAS instrument.

Cognitive, Affective, and Psychomotor Domain Evaluation: Participants submitted a video recording of their psychodynamic psychotherapy session with a patient diagnosed with BPD. The video was assessed using the PC-CAS instrument.

#### 2.5.3 Module implementation

Participants in the intervention group underwent structured instruction using the PP-BPD Learning Module for Indonesian Psychiatry Residents, delivered by the researchers over a three-month period, comprising 12 sessions.

Concurrently, the control group received a placebo intervention in a form of online module for a total of three sessions of online lectures with the last one focusing on implementing the theory into the practice of psychodynamic psychotherapy for BPD cases. Each session was conducted monthly and took about three months in total. The residents were blinded from the kind of intervention they were given. The raters were also blinded to the assignment of the groups. The raters were also blinded to the group in which the participants were assigned into.

#### 2.5.4 Post-intervention assessment (post-test)

Following the intervention period, post-test assessments were conducted for both groups, using the same evaluation instrument completed during the pre-intervention assessment. The intervention group completed the Indonesian version of the Kirkpatrick Level 1 questionnaire to assess their reactions to the module. Thematic analysis was then conducted by identifying the meaning units from participant answers, assigning codes into the meaning units, and categorizing them thematically.

## 3 Results

### 3.1 Participant demographic characteristics

The study was conducted between August 2023 and January 2024. A total of 34 participants were recruited, with one dropping out from the intervention group. The demographic characteristics of the 33 participants that completed the study are shown in **Table 1**.

**Table 1:** Demographic characteristics of participants (N = 33).

Participant characteristics	Intervention (n = 16)	Control (n = 17)
Age, year (mean ± SD)	34 ± 3.7	33,8 ± 4
Sex (n)		
Male	7	5
Female	9	12
Semester (n)		
6	9	10
7	7	7
Institution (n)		
Universitas Diponegoro		3
Universitas Hasanuddin		4
Universitas Indonesia		5
Universitas Udayana		5
Universitas Airlangga	1	
Universitas Gadjah Mada	4	
University of Sebelas Maret	5	
University of Padjadjaran	3	
Universitas Sumatera Utara	3	

### 3.2 Evaluation of learning outcomes of the PP-BPD learning module for Indonesian Psychiatry Residents

Based on the Shapiro-Wilk normality test and calculation of the coefficient of variance (COV), the pre-test PC-CAS scores in the intervention group, post-test PF-CAS scores in the intervention group, and post-test PC-CAS scores in both the control and intervention groups were normally distributed.

**Table 2** illustrate the pre-test and post-test scores of the multiple-choice questions. A significant difference was noted between the pre-test and post-test multiple-choice question scores in both the control and intervention groups. In addition, a significant difference was noted in the mean post-test multiple-choice question scores between the control and intervention groups. The mean post-test score in the intervention group was 7.2 points higher than that in the control group.

**Table 2:** Pre-test and post-test scores for multiple-choice questions.

	Control	Intervention	Mean Difference (CI 95%)	p^a^
	Mean ± SD	Mean ± SD		
*Pre-test*	43.4 ± 7.5	45.1 ± 8.2	1.7 (-3.9–7.3)	0.536
*Post-test*	51.3 ± 9.3	58.5 ± 9.3	7.2 (0.6–13.8)	0.033*
p^b^	0.001*	< 0.001*		

p^a^ = unpaired t-test.

p^b^ = paired t-test.

**Table 3** shows the mean increases from pre-test to post-test of the scores of multiple-choice questions, PF-CAS, and PC-CAS, and compares them between the intervention and control groups. The comparative analysis of pre-test and post-test multiple-choice question score differences revealed that the mean difference was 7.9 ± 8.4 in the control group, while it was 13.4 ± 8.2 in the intervention group. Based on the unpaired t-test analysis, the difference in the intervention group was 5.5 points higher than that in the control group; however, this difference was not statistically significant (p = 0.067). Given the significance value of α = 0.05, observed power was found to be 0.451.

**Table 3:** Comparative analysis of pre-test and post-test multiple-choice question, PF-CAS, and PC-CAS score differences.

	Mean ± SD	Mean difference (CI 95%)	p
Multiple Choice Questions			
Control	7.9 ± 8.4	5.5 (-0.4–11.4)	0.067^a^
Intervention	13.4 ± 8.2		
PF-CAS			
Control	13.7 ± 13.3	39.1 (28.0–50.2)	< 0.001*^b^
Intervention	52.8 ± 17.7		
PC-CAS			
Control	16.5 ± 18.7	8.4 (-3.3–19.9)	0.154^a^
Intervention	24.9 ± 13.3		

a = unpaired t-test.

b = Mann-Whitney test.

**Figure 2:**
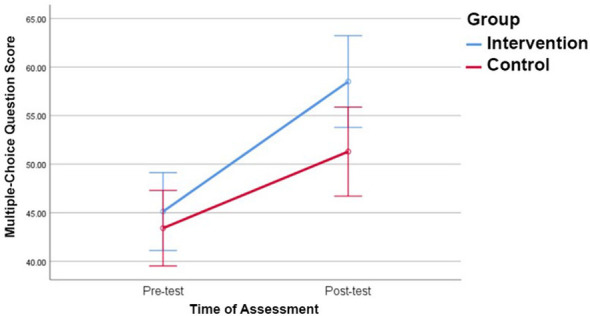
Two-way repeated measures ANOVA analysis of multiple-choice questions scores.

As seen in **Figure 2**, two-way repeated-measures ANOVA revealed an increase in multiple-choice question scores in both the control and intervention groups. However, the difference between the two time points (pre- and post-test) was not statistically significant (p = 0.067).

**Table 4** illustrate the pre-test and post-test scores of the PF-CAS and PC-CAS. A significant difference was noted between the pre- and post-test PF-CAS scores in both the control and intervention groups. Additionally, there was a significant difference between the median post-test PF-CAS scores of the control and intervention groups, with the mean post-test PF-CAS score in the intervention group being 31.2 points higher than that in the control group (p < 0.001).

**Table 4:** Pre-test and post -test scores for PF-CAS and PC-CAS.

	Control		Intervention		Mean difference (CI 95%)	p
	Mean ± SD	Median (min–max)	Mean ± SD	Median (min–max)		
PF-CAS						
Pre-test	46.7 ± 20.8	44.4 (11–94)	38.9 ± 15.3	41.6 (11–66)	7.8 (-20.9–5.2)	0.435^a^
Post-test	60.5 ± 23.4	61.1 (22–100)	91.7 ± 11.2	100 (66–100)	31.2 (18.1–44.4)	< 0.001*^a^
p		0,002*^b^		0,001*^b^		
PC-CAS						
*Pre-test*	63.3 ± 18.3	60.6 (36–93)	44.2 ± 7.9	43.4 (27–58)	-19.1 (-29.2– -8.9)	0.001*^a^
*Post-test*	79.9 ± 13.2	72.7 (60–100)	69.1 ± 10.7	69.7 (51–90)	-10.8 (-19,4– -2,2)	0.015*^c^
p^b^		0.002*^b^	0.001*^d^			

a = Mann-Whitney test.

b = Wilcoxon test.

c = unpaired t-test.

d = paired t-test.

Analysis of the differences of mean increases from pre-test to post-test of the scores of PF-CAS and PC-CAS between the intervention and control groups is shown in **Table 3**. The difference between pre-test and post-test PF-CAS scores was 13.7 ± 13.3 in the control group, while it was 52.8 ± 17.7 in the intervention group. The Mann-Whitney test showed that the increase in PF-CAS scores in the intervention group was 39.1 points higher than that in the control group, and this difference was statistically significant (p < 0.001). The effect size of the difference between the two medians was Cliff’s delta = 0.889, indicating a large effect size. Power analysis showed that observed power was 1.00 given the significance value of α = 0.05.

**Figure 3:**
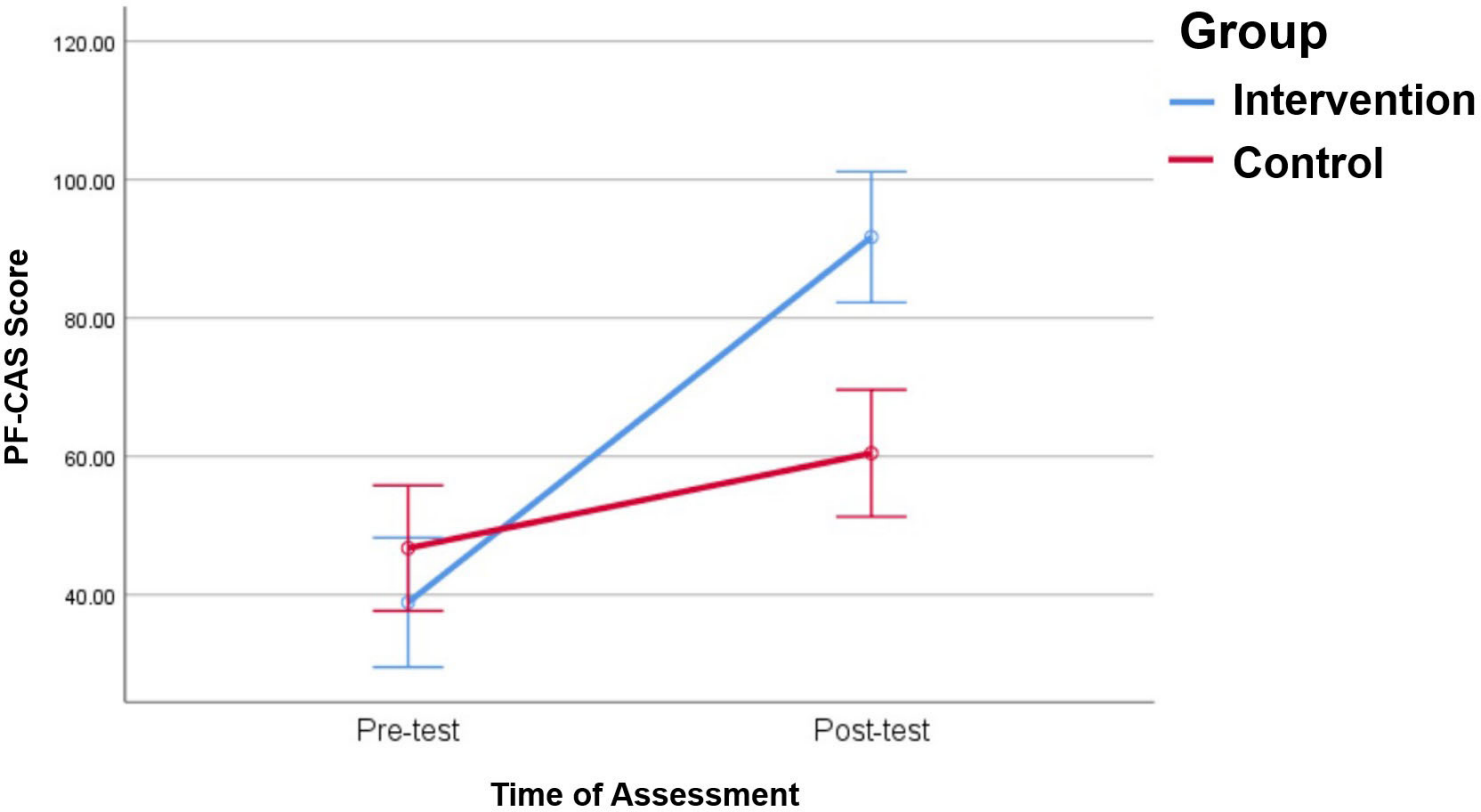
Two-way repeated measures ANOVA analysis of PF-CAS scores.

**Figure 3** shows the two-way repeated-measures ANOVA analysis of the PF-CAS scores. The analysis revealed that the increase in PF-CAS scores in both the control and intervention groups between the pre-test and post-test was statistically significant (p < 0.001).

Based on the Mann-Whitney test, there was a significant difference between the median pre-test PC-CAS scores of the control and intervention groups (p = 0.001), with the control group scoring 19.1 points higher than the intervention group. In the post-test, the control group still had higher PC-CAS scores than the intervention group, with a mean difference of 10.8 points, according to the unpaired t-test (p = 0.015).

The difference between pre-test and post-test PC-CAS scores in the control group was 16.5 ± 18.7, while in the intervention group, it was 24.9 ± 13.3. A comparative analysis of the pre- and post-test PC-CAS differences showed numerically greater improvement in the intervention group by 8.4 points higher than the control group. However, this difference was not statistically significant (p = 0.154).

**Figure 4:**
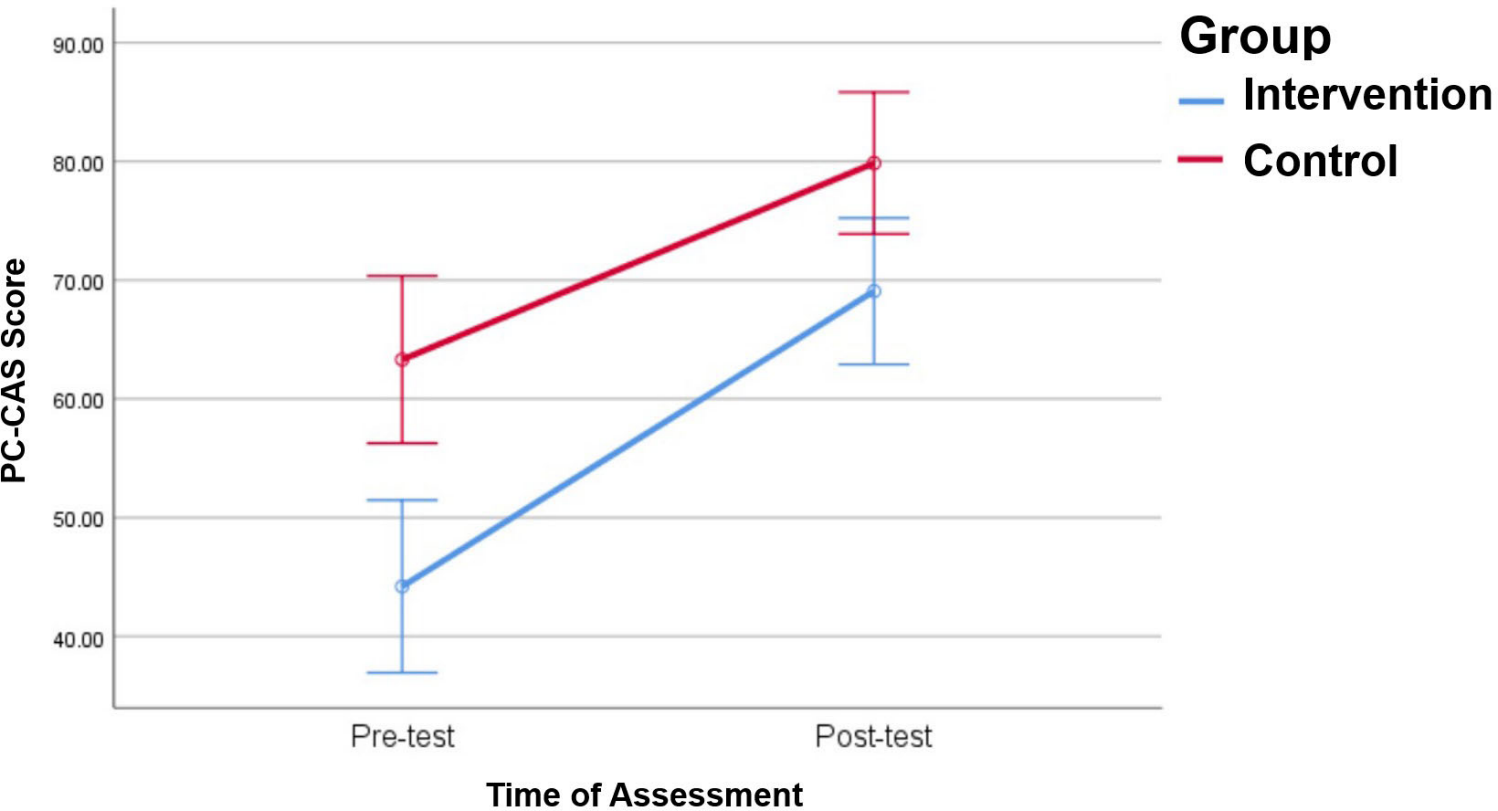
Two-way repeated measures ANOVA analysis of PC-CAS scores.

The two-way repeated-measures ANOVA analysis of the PC-CAS scores can be seen on **Figure 4**. Two-way repeated measures ANOVA found that the increase in PC-CAS scores in both the control and intervention groups between the pre-test and post-test assessments was not statistically significant (p = 0.154). This might be due to the small sample size which made the result to be underpowered, as shown by the power analysis. Power analysis showed that observed power was 0.294 given the significance value of α = 0.05.

Additionally, we performed an ANCOVA analysis to examine the difference in the PC-CAS scores difference from pre-test to post-test across both groups while controlling the baseline PC-CAS scores as a covariate. After adjusting baseline pre-test differences, we found the effect of intervention groups on PC-CAS score differences from pre-test to post-test was not statistically significant (p = 0.180). Furthermore, baseline PC-CAS scores were significantly related to PC-CAS score differences from pre-test to post-test (p = 0.000).

### 3.3 Participant reactions to the PP-BPD learning module for Indonesian Psychiatry Residents

Based on the Indonesian version of the Kirkpatrick Level 1 Questionnaire, participants provided positive feedback regarding the PP-BPD Learning Module for Indonesian Psychiatry Residents. All participants (100%) stated that their learning experiences were enriched by the knowledge, experience, and examples shared by the facilitator. Approximately 75% of the participants felt comfortable with the session duration, whereas 25% responded neutrally. The content analysis of the participant feedback produced five themes of the most important aspects learned from the module (**Table 5**), as well as two main suggestions for the module (**Table 6**).

**Table 5:** Participants’ most valued learning points from the PP-BPD learning module.

Theme	Subtheme	Quotations
1. Psychoanalysis theory in BPD patients	Psychoanalysis theory: self psychology, object relation, and attachment	*“Before I enrolled in this module, I had a hard time understanding and integrating basic theories (Freud, Melanie, Bowlby, Kohut, etc) in making assessment and formulation, and doing psychodynamic psychotherapy. I could not even remember the names of the theories.” (Resident-8)*
	Understanding the process of parenting and psychodynamic theory in BPD patients	*“Understanding how parenting process is involved in BPD, therefore making psychodynamic psychotherapy will help us managing BPD patients.” (Resident-15)*
2. Psychodynamic formulation	Steps on how to make psychodynamic assessment	*“Understanding the process of making a psychodynamic assessment.” (Resident-9)*
	Components of psychodynamic formulation	*“Steps on how to write a good and complete psychodynamic formulation.” (Resident-12)*
	Assessing and making psychodynamic formulation to assist the therapists in understanding and managing BPD patients	*“… Therefore making psychodynamic formulation in patients will help us managing BPD patients.” (Resident-15)*
3. Process notes of psychodynamic psychotherapy	Understanding how to use the appropriate technique of conducting psychodynamic psychotherapy by creating process notes	*“When learning the different kinds of psychodynamic psychotherapy techniques by creating process notes, I reflected on my past practice and realized I had been just as confused as my patients which might hinder my ability in helping them or might even create an alliance rupture.” (Resident-8)*
	Being able to reflect on the psychotherapy practice through process notes discussion of psychodynamic psychotherapy	*“Making psychotherapy process notes could help reflect on things that needed improvement.” (Resident-14)*
4. Skills of conducting psychodynamic psychotherapy in BPD patients	Understanding the techniques of psychodynamic psychotherapy and implementing them accordingly	*“Conducting psychodynamic psychotherapy requires a lot of practice so that the responses given are appropriate to the patients’ conditions.” (Resident-14)*
	Learning, understanding, and tolerating resistance, transference, and countertransference to manage sessions better	*“Learning about transference, countertransference, resistance, alliance rupture, and how to manage them during therapy session.” (Resident-13)*
	Understanding the concept of working through	*“How as a therapist, I should be able to do ‘holding and containment’ in each session to manage both my patient’s and my own emotions” (Resident-15)*
5. The role of therapist in managing BPD patients	Understanding the needs of BPD patients	*“As a therapist, I should be willing to listen to patients’ stories and questions.” (Resident-7)*
	Helping BPD patients to process their unconscious materials to help patients gradually achieve better function and interpersonal relationships	*“…should understand what the patient needs by processing their unconscious problems so they can continue to live a better life.” (Resident-5)*
	Understanding that psychodynamic psychotherapy in BPD patients takes a great level of patience and time due to the complexity of their problems	*“Patients are difficult to manage, not because of the therapist’s incompetence, rather than the complexity of their problems.”* *(Resident-3)*

**Table 6:** Participants’ feedback toward PP-BPD learning module.

Theme	Subtheme	Quotations
Theme 1: Learning Materials	Adding the variety of case illustrations, protocols, and psychotherapy practice videos	*- ****“*** *I think the module could include more case illustrations, as it might enrich its content.” (Resident-8)*
	Supplementing the textbook with multiple-choice questions as formative exercises	- *“It may be worth considering the addition of MCQs and their answers in the module book.”* (*Resident*-*12*)
Theme 2: Teaching Methods	Adding more sessions for psychodynamic psychotherapy practice	- *“Providing more time for residents to practice as future therapists (longer module duration and more interview practice assignments, allowing for more evaluations).”* (*Resident-13*)
	Increasing supervision during psychodynamic psychotherapy practice sessions	- *“Regular psychotherapy interview practice, based on the module guidelines and accompanied by a supervisor, is needed.”* (*Resident-10*)

## 4 Discussion

The PP-BPD Learning Module for Indonesian Psychiatry Residents was the first instructional model that specifically taught psychodynamic psychotherapy for BPD cases among Indonesian psychiatry residents. Based on the analysis of the multiple-choice instrument scores, no significant difference was noted in the mean pre-test scores between the control and intervention groups. However, a significant difference was observed in the mean post-test scores, with the intervention group’s post-test scores being 7.2 points higher than those of the control group. When analyzing the difference between pre- and post-test scores, the intervention group showed an increase of 5.5 points more than the control group, although this difference was not statistically significant.

A positive cognitive domain shift was observed in the intervention group; however, the difference was not statistically significant. This can be explained by the fact that both the intervention and control groups received lectures that provided equal learning opportunities for all participants. The module and textbook did not incorporate formative evaluation methods for knowledge acquisition, such as quizzes or multiple-choice questions, between module sessions. Multiple-choice questions assess the cognitive aspects of a broad topic, making it challenging for participants to master the knowledge required to answer them.

Analysis of the PC-CAS scores showed that the median posttest PF-CAS score in the intervention group was 31.2 points higher than that in the control group. The difference in pre-test and post-test PC-CAS scores in the intervention group was 39.1 points higher than that in the control group, and this difference was statistically significant. These results indicate that participants who completed the PP-BPD Learning Module for Indonesian Psychiatry Residents demonstrated greater competence improvement in both the cognitive and affective domains compared to those in the control group.

One of the learning tasks in this module is the development of psychodynamic formulations. The participants were provided with supporting information and sufficient examples to develop psychodynamic formulations and discuss them with the tutor. This teaching and learning process served as positive feedback, contributing to competency development in both the cognitive and affective domains for psychodynamic formulation.

The mean post-test PC-CAS score of the control group was significantly higher than that of the intervention group. Although we had strived for equal baseline scores between the group by employing stratified random sampling, the control group scored significantly higher at pre-test on PC-CAS. Both groups experienced improved PC-CAS scores, with the intervention group showing an 8.4-point higher increment than the control group. However, this difference was not statistically significant. The difference in the pre-test and post-test PC-CAS scores was 16.5 points in the control group and 24.9 points in the intervention group. This suggests that the intervention group demonstrated greater cognitive, affective, and psychomotor domain improvements than the control group. The lack of statistical significance might be due to insufficient amount of practice, brief duration of training, or baseline score imbalance on the pre-test scores.

Miller’s clinical competency development pyramid consists of four levels: acquiring knowledge about competence (“knows”), understanding how to apply that competence (“knows how”), demonstrating the ability to perform the competence (“shows how”), and practicing the competence in real-life settings (“does”). The third level (“shows how”) can only be evaluated in a simulation setting, while the fourth level (“does”) can only be assessed through workplace-based evaluation. Both the third and fourth levels involve a combination of cognitive, affective, and psychomotor learning domains (20).

Performing psychodynamic psychotherapy for patients with BPD falls under the fourth level and requires complex skills. Research suggests that the cognitive, affective, and psychomotor domains are closely interrelated. The affective domain serves as a foundation for improving cognitive and psychomotor skills by enhancing motivation for in-depth learning (21–23). Achieving fourth-level competencies requires more time and frequent practice (20, 24). The cognitive, affective, and psychomotor domains are interconnected in developing competency in psychodynamic psychotherapy for patients with BPD. The affective domain plays a crucial role in enhancing learning across all three domains, ultimately shaping competent therapists. As reflected in the PC-CAS evaluation, the competency in performing psychodynamic psychotherapy for patients with BPD improved in the intervention group. As seen through the PF-CAS assessment, although the difference was not statistically significant, the results remained promising, as the intervention group had already shown strong affective development. This suggests that the module successfully fostered affective behavioral changes, which would eventually be followed by improvements in the psychomotor domain.

The practical psychodynamic psychotherapy training for patients with BPD in this module consisted of seven sessions, including five feedback sessions in which participants discussed video recordings and processed notes of psychodynamic psychotherapy sessions. These learning activities enhanced psychodynamic psychotherapy competency for BPD patients but were not sufficient to result in a statistically significant improvement. Additional training sessions may be required for participants to practice and receive feedback before they undergo a summative assessment in the final evaluation. Participants might also benefit from direct supervision while undergoing psychodynamic psychotherapy.

Formative assessments were conducted through discussions and feedback on written psychodynamic formulation reports, video recordings of psychodynamic psychotherapy sessions, and process notes of psychodynamic psychotherapy sessions. These formative assessments provided participants with feedback for improvement and competency development, while motivating them to continue learning. Burgess et al. supported this approach, and Ismail et al. found that formative assessments with feedback increased academic motivation and self-regulation skills and reduced test anxiety (25, 26). In this module, formative assessments focus on formulating a psychodynamic formulation and the practical competency of performing psychodynamic psychotherapy. Multiple-choice questions are not included in the formative evaluation. Textbook materials were developed as learning resources for preparing multiple-choice summative evaluations. Previous studies on psychotherapy training models for BPD, such as those by Unruh et al., Deranja et al., and Haliburn et al., did not discuss formative assessments during training (11, 27, 28). The inclusion of formative assessments of the skills required to formulate a psychodynamic formulation and the practical competency of performing psychodynamic psychotherapy are unique strengths of the PP-BPD learning module for Indonesian psychiatric residents.

Supervision is a crucial component of psychotherapy. According to the Accreditation Council for Graduate Medical Education (ACGME), three types of supervision exist: direct, indirect, and oversight/delay. In this module, oversight supervision was implemented by reviewing the recorded psychodynamic psychotherapy sessions conducted by the participants. Video supervision has become common practice in psychiatric residency training. Tutors can use the pause feature to discuss a trainee’s psychotherapy interactions with patients. This allows trainees to review, reflect on, and discuss the conducted psychotherapy processes (29, 30).

The PP-BPD Learning Module for Indonesian Psychiatry Residents can be further developed to incorporate direct supervision through video conferencing. According to Kivlighan et al., direct supervision has a greater effect than oversight supervision (31). Gammon et al. reported that psychiatric residents who received videoconferencing supervision experienced the same level of satisfaction as those who received in-person supervision. In Gammon’s study, all residents and supervisors met in person, establishing relationships built on mutual trust and respect (32). Supervisors can implement several strategies to enhance the quality of videoconferencing supervision, including setting a clear focus on the psychotherapy session, maintaining respectful and professional communication such as using appropriate nonverbal cues, and being flexible in utilizing other technological modalities. In addition, the technology used should ensure high-quality audio (32, 33). In the implementation of the PP-BPD Learning Module for Indonesian Psychiatry Residents within the curriculum, formative assessment can be conducted through both direct and oversight supervision as part of the Direct Observation of Procedural Skills (DOPS) using the PC-CAS instrument. Further development and implementation of this module is to be adapted for international curricula in other psychiatry residency programs.

Based on the Kirkpatrick Level 1 questionnaire, the module participants responded positively to the implementation of the learning module and stated that the module significantly contributed to their competency development as therapists. None of the participants reported any negative effects, such as discomfort or boredom during the module. A positive reaction to an educational model indicated that participants appreciated the training module and were more likely to continue learning in the future (34).

Based on a thematic analysis of participants’ responses, the most important aspects learned from the module included: (1) psychoanalytic theory of BPD, (2) psychodynamic formulation, (3) psychodynamic psychotherapy process notes, (4) psychodynamic psychotherapy skills for patients with BPD, and (5) the therapist’s role in managing patients with BPD. Learning about psychoanalytic theory and psychodynamic formulation helped psychiatric residents deepen their understanding of BPD patients’ psychodynamics and applied psychoanalytic theory to develop case formulations. Discussions of psychodynamic psychotherapy process notes improved their ability to select more therapeutic interventions while interacting with patients. Participants reported a better understanding of their role as therapists in managing patients with BPD, recognizing that the complexity of these cases did not imply a lack of therapists’ competence. This suggests that the learning module provided meaningful benefits, enhancing the participants’ confidence in treating BPD cases with psychodynamic psychotherapy. Keuroghlian et al. also found that specialized training in BPD reduced the perception among psychiatric residents that BPD was an untreatable disorder (35).

Participants suggested increasing the variety of case illustrations, adding more sessions discussing psychodynamic psychotherapy process notes and video recordings, incorporating multiple-choice questions into the textbook as formative exercises, increasing the number of psychodynamic psychotherapy practice sessions, and providing more supervision during practice sessions.

The limitations in this study are acknowledged. This study had a small sample size and a limited statistical power when analyzed. Baseline imbalance in assessing practical skills by PC-CAS might affect the statistical power of the significance value within the results. This study was also confined within the country.

## 5 Conclusions

The Psychodynamic Psychotherapy for Borderline Personality Disorder Learning Module significantly enhanced residents’ competencies in formulating a psychodynamic formulation and performing psychodynamic psychotherapy with particularly strong gains in the cognitive and affective domains. While improvements in practical skills showed a promising trend albeit not statistically significant, participant feedback highlighted the value and feasibility of this module. Further refinement with expanded supervision and practice opportunities may strengthen its impact and support its integration into residency training curricula. Long-term follow up to assess skill sustainability in residents following the module and the clinical impact on patient care should be furtherly studied.

## Data Availability

The datasets presented in this article are not readily available because the participants did not give consent to the public sharing of the data. Requests to access the datasets should be directed to ptrn1010@yahoo.com.
