# Supplementary material for: Enhancing psychiatry education: effectiveness of a psychodynamic psychotherapy module for borderline personality disorder for psychiatry residents
Source: Front Psychiatry. 2026 Jul 2;17:1712435. doi: 10.3389/fpsyt.2026.1712435 (PMC13372892; doi:10.3389/fpsyt.2026.1712435)
Supplement: Supplementary file 1 [file Table1.docx]

**Supplementary Material 1. Summary of the Results of Kirkpatrick level 1 Indonesian Version, in Indonesian Language**

| **Butir Kuesioner** | **Nilai 1 (STS)* (n)** | **Nilai 2 (TS)* (n)** | **Nilai 3 (N)* (n)** | **Nilai 4 (S)* (n)** | **Nilai 5 (SS)* (n)** |
| --- | --- | --- | --- | --- | --- |
| 1. **Saya memahami sasaran pembelajaran modul** | **0** | **0** | **0** | **5** | **11** |
| 1. **Saya memperoleh pengetahuan dan keterampilan yang sesuai dengan sasaran pembelajaran modul** | **0** | **0** | **0** | **4** | **12** |
| 1. **Saya paham apa yang diharapkan dari saya sebagai seorang terapis setelah mengikuti modul ini** | **0** | **0** | **0** | **6** | **10** |
| 1. **Saya mendapati materi pembelajaran (contoh: *slide* kuliah, dst) mudah dipahami dan dijalankan** | **0** | **0** | **0** | **2** | **14** |
| 1. **Saya mendapati tingkat kompleksitas dan detail dari materi sesuai dengan latar belakang dan pengalaman saya** | **0** | **0** | **1** | **6** | **9** |
| 1. **Materi pembelajaran, termasuk sumber materi lainnya, sangat penting bagi keberhasilan saya sebagai terapis** | **0** | **0** | **0** | **4** | **12** |
| 1. **Saya dapat mengaplikasikan apa yang saya pelajari di modul ini dalam peran saya sebagai seorang terapis** | **0** | **0** | **0** | **8** | **8** |
| 1. **Saya sudah mendapatkan pengetahuan dan keterampilan yang dibutuhkan untuk menjadi terapis yang baik melalui modul ini** | **0** | **0** | **0** | **7** | **9** |
| 1. **Saya mengerti di mana saya akan mencari jawaban untuk pertanyaan yang timbul dalam peran saya sebagai terapis** | **0** | **0** | **0** | **9** | **7** |
| 1. **Pembelajaran saya diperkaya oleh pengetahuan fasilitator** | **0** | **0** | **0** | **0** | **16** |
| 1. **Pembelajaran saya diperkaya oleh pengalaman dan contoh yang dibagikan fasilitator** | **0** | **0** | **0** | **2** | **14** |
| 1. **Saya mampu menyimak secara baik selama sesi pelatihan** | **0** | **0** | **2** | **9** | **5** |
| 1. **Saya mendapati bahwa diri saya mudah untuk terlibat secara aktif selama pelatihan berlangsung** | **0** | **0** | **3** | **5** | **8** |
| 1. **Saya memiliki banyak kesempatan untuk bertanya dan memperoleh jawaban atas pertanyaan saya dari fasilitator selama sesi pelatihan** | **0** | **0** | **1** | **4** | **11** |
| 1. **Saya memiliki banyak kesempatan untuk melatih atau memperagakan keterampilan yang telah saya pelajari selama sesi berlangsung** | **0** | **0** | **0** | **8** | **8** |
| 1. **Saya merasa nyaman dengan ritme dan kecepatan sesi pelatihan** | **0** | **0** | **1** | **8** | **7** |
| 1. **Saya merasa nyaman dengan durasi berjalannya sesi** | **0** | **0** | **4** | **5** | **7** |
| 1. **Media pembelajaran menyenangkan dan kondusif untuk proses pembelajaran** | **0** | **0** | **0** | **8** | **8** |

***STS=Sangat Tidak Setuju, TS=Tidak Setuju, N=Netral, S=Setuju, SS=Sangat Setuju**
