# Supplementary material for: Enhancing psychiatry education: effectiveness of a psychodynamic psychotherapy module for borderline personality disorder for psychiatry residents
Source: Front Psychiatry. 2026 Jul 2;17:1712435. doi: 10.3389/fpsyt.2026.1712435 (PMC13372892; doi:10.3389/fpsyt.2026.1712435)
Supplement: Supplementary file 2 [file Table2.docx]

**Supplementary Material 1. Outline of the PP-BPD Learning Module**

| **Submodule** | **Quotations** |
| --- | --- |
| 1. Borderline Personality Disorder | - Definition - Diagnostic criteria - Psychopathology and biopsychosocial model - Neurobiological factors - Psychosocial impact - Clinical management and prognosis - Psychodynamic models of borderlinepersonality disorder (ego psychology, object relations, self psychology, and attachment theory) - Psychoeducation for the patient and the family |
| 1. Basic Principles of Psychodynamic Psychotherapy | - Basic theories of psychoanalysis - Basic principles of psychodynamic psychotherapy - Neuroscience aspects of psychodynamic psychotherapy - Initial assessment and indications - Psychodynamic diagnosis - Psychodynamic formulation - Goals of psychodynamic psychotherapy - Psychodynamic interventions |
| 1. The Practice of Psychodynamic Psychotherapy for Patients with Borderline Personality Disorder | - Beginning the therapy session (informed consent, negotiating goals, determining limits of therapy) - Working through - Strategies for maintaining the therapeutic alliance, rupture of the therapeutic alliance, and impasse - Managing emotional dysregulation - Managing resistance, transference, countertransference - Evaluating the progress of the therapy - Termination |
